# The other giant: functional significance and divergence of Type I MADS-box genes in the evolution of land plants

**DOI:** 10.1093/jxb/erag134

**Published:** 2026-03-10

**Authors:** Yichun Qiu, Claudia Köhler

**Affiliations:** Department of Plant Reproductive Biology and Epigenetics, Max Planck Institute of Molecular Plant Physiology, Potsdam 14476, Germany; Department of Plant Reproductive Biology and Epigenetics, Max Planck Institute of Molecular Plant Physiology, Potsdam 14476, Germany; Department of Plant Biology, Uppsala BioCenter, Swedish University of Agricultural Sciences and Linnean Centre for Plant Biology, Uppsala 75007, Sweden; University College Dublin, Ireland

**Keywords:** Endosperm, evo-devo, gametophyte, gene duplication, land plant, M-type, MADS-box transcription factor, MIKC*-type, Type I

## Abstract

Angiosperms represent the most abundant and diverse lineage of land plants, and their evolutionary success is closely linked to major reproductive innovations, including the origin of flowers and the embryo-nourishing endosperm. Many of the genes underlying these innovations belong to the MADS-box transcription factor family. While the functions of MIKC^C^-type MADS-box genes in floral development are well established, this review focuses on the other two lineages, M-type and MIKC*-type genes, and synthesizes recent advances in the understanding of their functional roles and evolutionary histories. M-type genes are key regulators of female gametophyte and endosperm development, indicating that two hallmark features of angiosperms were promoted by distinct MADS-box gene classes. MIKC*-type genes govern pollen development and are phylogenetically more closely related to M-type genes; together, they form the plant-specific Type I clade, which primarily regulates gametophytic programmes. This contrasts with the Type II clade, comprising MIKC^C^-type genes that diversified to control sporophytic development. Both Type I and Type II clades originated from plant-specific duplications of ancestral MEF2-like genes. Through extensive lineage-specific expansion and diversification, these MADS-box transcription factors have played a central role in plant terrestrialization by integrating stress responses with reproductive development and the patterning of progressively complex body plans.

## Teaser

This review synthesizes current knowledge of land plant Type I MADS-box transcription factors, highlighting their evolutionary history and divergence patterns, and the key roles they play in diverse reproductive programmes.

## Introduction

MADS-box genes are a famous and intriguing family of eukaryotic transcription factors (TFs) that regulate diverse biological processes in animals, fungi, plants, and protists (Messenguy and Dubois, 2003). The name MADS derives from the four founding members, Minichromosome maintenance 1 (Mcm1) from *Saccharomyces cerevisiae*, AGAMOUS from *Arabidopsis thaliana*, DEFICIENS from *Antirrhinum majus*, and Serum response factor (SRF) from *Homo sapiens* (Schwarz-Sommer *et al*., 1990). Ever since this gene family was discovered, MADS-box TFs have been a subject of intense investigations over decades.

The MADS-box gene family in land plants is traditionally divided into MIKC-type and M-type based on domain architecture (Alvarez-Buylla *et al*., 2000). MIKC-type TFs typically contain the canonical MADS (M) domain followed by the Intervening (I), Keratin-like (K), and C-terminal (C) domains, whereas in M-type TFs the MADS domain is the only well-characterized conserved region. M-type and MIKC-type are also frequently referred to as Type I and Type II MADS-box TFs, respectively. These two groups have both undergone substantial expansion during the evolution of land plants (Nam *et al*., 2003; Gramzow and Theißen, 2013; Qiu *et al*., 2023). The functional evolution of MADS-box genes has greatly contributed to the success of land plants. In particular, expansions of the MADS-box gene family are associated with increased body plan complexity, providing the genetic toolkit for many developmental novelties (Theißen *et al*., 1996, 2000; Kaufmann *et al*., 2005; Thangavel and Nayar, 2018). Angiosperms, the most abundant and diverse lineage of land plants, owe much of their evolutionary success to innovations in sexual reproduction, including the evolution of flowers and fruits. Extensive studies have established that the duplication and diversification of Type II MADS-box genes were central to the origins of these structures (Ng and Yanofsky, 2001; Kaufmann *et al*., 2005; Theißen *et al*., 2016). Through interactions mediated by the plant-specific K domain, many MIKC-type TFs assemble into floral quartet-like complexes, higher-order regulatory tetramers that specify floral organ identity (Smaczniak *et al*., 2012; Theißen *et al*., 2016).

In contrast to the popular Type II, Type I MADS-box genes were not discovered until annotated in the first angiosperm genome of *A. thaliana* (Alvarez-Buylla *et al*., 2000). Early research into this group was hindered by their low and spatially restricted expression patterns, as well as by their high copy number and extensive functional redundancy (Kofuji *et al*., 2003; Parenicová *et al*., 2003). Over the past 25 years, however, the surge in genomic resources and the development of new experimental technologies have enabled more comprehensive analyses of Type I genes. In this review, we summarize current advances in understanding the evolution, diversification, and biological roles of Type I MADS-box transcription factors.

### A new model of the evolution of plant MADS-box genes

#### Two rounds of ancient gene duplication established the major MADS-box lineages in land plants

While land plants have two major types of MADS-box genes, it is noteworthy that animal genomes also possess two primary MADS-box gene types, SRF and Myocyte Enhancer Factor-2 (MEF2). An influential early hypothesis proposed that SRF and MEF2 originated from an ancient gene duplication pre-dating the diversification of eukaryotes, and that plant Type I genes were related to SRF, whereas plant Type II genes were orthologous to MEF2 (Alvarez-Buylla *et al*., 2000). This model shaped thinking about MADS-box gene evolution for decades. However, Alvarez-Buylla *et al*. (2000) already noted that the orthology between plant Type I TFs and animal SRF required stronger evidence, a concern shared by others (Kaufmann *et al*., 2005). The first phylogeny, constrained by limited sequence availability, lacked the resolution needed to robustly infer relationships among highly divergent sequences, later shown to be a hallmark of plant M-type TFs (Nam *et al*., 2004; Gramzow and Theißen, 2013). Despite these caveats, the initial model was often taken for granted, as many studies classified plant Type I (M-type) and Type II (MIKC-type) genes based solely on domain structures and then reconstructed phylogenies for each type separately, rather than testing the broader evolutionary relationships.

A robust re-evaluation required phylogenies that combined adequate ingroup sampling of plant MADS-box genes with appropriate outgroups from non-plant eukaryotes. Leveraging newly available sequences across the eukaryotes, a more comprehensive phylogeny substantiated the ancient eukaryote-wide SRF and MEF2 duplication proposed by Alvarez-Buylla *et al*. (2000), and moreover, demonstrated that the two major plant MADS-box types were plant-specific duplicates, both tracing back to an ancestral MEF2-like gene, whereas SRF-type genes were absent in the most recent common ancestor of all green lineages (Qiu *et al*., 2023). Thus, plant Type I and Type II MADS-box genes diverged far more recently than previously assumed. This early duplication of a MEF2-derived ancestral gene produced the precursors of the modern Type I and Type II clades, setting the stage for the subsequent extensive expansion of the plant MADS-box gene family.

Within MIKC-type TFs, the MIKC*-type is distinguishable from the ‘classic’ MIKC^C^-type (Henschel *et al*., 2002). Although they were grouped together because of their shared plant-specific K domain, early phylogenies often disagreed about the placement of the MIKC*-type genes (Alvarez-Buylla *et al*., 2000; De Bodt *et al*., 2003a, b; Kofuji *et al*., 2003; Martinez-Castilla and Alvarez-Buylla, 2003; Nam *et al*., 2004). Building on the updated MEF2-origin model, a broader survey across diverse land plant lineages clarified the deeper relationships among the three major MADS-box gene types (Qiu *et al*., 2024). It revealed that the precursor of the MIKC^C^-type diverged first, followed by a subsequent duplication that split the MIKC*-type and M-type precursors. Consequently, the MIKC*-type and M-type lineages are more closely related, together constituting an updated Type I clade (Qiu *et al*., 2024).

#### Structural evolution of MADS-box transcription factors prior to the divergence of land plants

Understanding the functional diversification of MADS-box genes requires a detailed understanding of how the structures of land plant MADS-box genes have diverged. With the growing availability of genomic and transcriptomic data from charophytes, the paraphyletic sister groups of land plants including the closest relatives of land plants, Zygnematophyceae (Hess *et al*., 2022), an increasing number of charophytic MADS-box gene sequences are shedding light on the early evolution of land plant MADS-box genes. The K domain is broadly detected in charophytic, but consistently absent from chlorophytic MADS-box TFs, supporting the view that MIKC-type MADS-box genes originated in the streptophyte lineage (Tanabe *et al*., 2005; Nishiyama *et al*., 2018; Gramzow *et al*., 2023, Preprint; Rümpler *et al*., 2023; Feng *et al*., 2024; Han *et al*., 2025). Charophytic MIKC-type genes likely retain the ancestral organization of the K-box, typically comprising four exons (Rümpler *et al*., 2023). In contrast, the three major land plant MADS-box gene types exhibit lineage-specific modifications to this ancestral structure: MIKC^C^-type genes lack the first exon and contain a duplication of the last exon; MIKC*-type genes show duplications of the first and second exons (Rümpler *et al*., 2023); and M-type genes have lost the entire K-box (Qiu *et al*., 2023).

Because each charophyte species possesses only one or up to six MADS-box genes, it has been thought that the major split between Type I and Type II MADS-box genes, and thus the formation of the MIKC^C^-, MIKC*-, and M-type lineages, occurred around the origin of land plants (Tanabe *et al*., 2005; Nishiyama *et al*., 2018; Gramzow *et al*., 2023, Preprint; Rümpler *et al*., 2023; Feng *et al*., 2024). A recent phylogeny, however, challenged this view by showing that charophytic MIKC-type TFs cluster with the land plant MIKC^C^-type clade (Han *et al*., 2025). This unexpected result suggests an alternative scenario in which the divergence of MIKC^C^-type and MIKC*-type genes occurred before the emergence of all streptophytes (Han *et al*., 2025). While this refined timeline for the ancient MIKC-type duplication is made possible by the recent surge in algal sequences, it rests on the assumption that at least five independent losses of the Type I lineage, including MIKC*-type and M-type orthologues, occurred across the major charophyte classes. Another, more parsimonious explanation is that the clustering of charophytic MIKC-type genes with land-plant MIKC^C^-type genes reflects the greater sequence conservation of the MIKC^C^-type lineage compared to MIKC*-type genes, rather than true orthology. Further sampling and improved models of sequence evolution are required for resolving the timing and consequence of these ancient gene divergences.

**Fig. 1.**
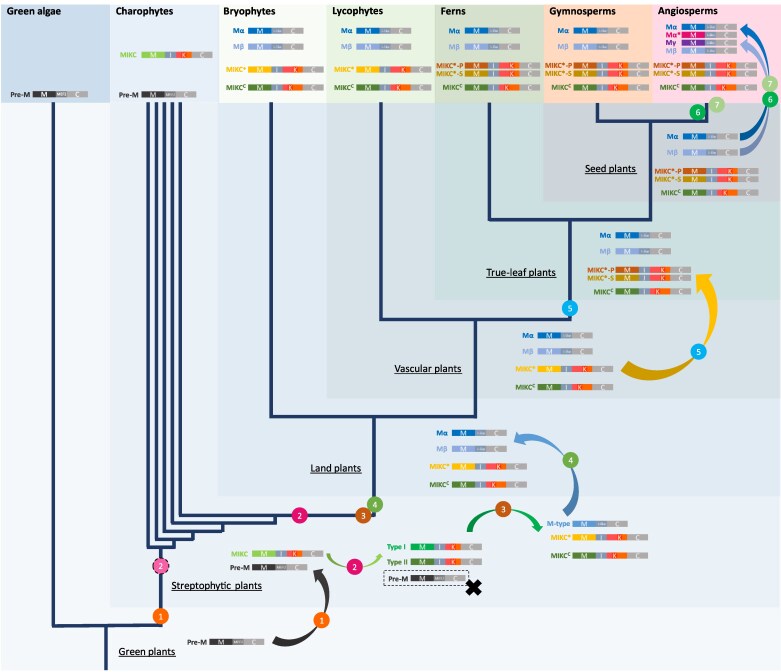
Evolution of MEF2-derived MADS-box transcription factors (TFs) in green plants. The top panel shows the main subfamilies present in each extant plant lineage, focusing on the origin and diversification of Type I (M-type and MIKC*-type) TFs without detailing the further duplication within Type II (MIKC^C^-type) TFs. The arrows mark the sequential gene duplication events, with the numbered circles labelling the corresponding branches inferred by phylogenetic timing. The ancestral gene in the most recent common ancestor (MRCA) of green plants resembled the MEF2-type TF, with the MADS-domain (canonical definition) followed by the MEF2 domain and a non-conserved C terminus. This structure was inherited in green algae. Prior to the MRCA of streptophytes, Duplication ① gave rise to two paralogous types which were inherited in charophytes, one of which retained the ancestral structure as those in green algae, and the other acquired the plant-specific K domain and therefore evolved to be the MIKC-type (Gramzow *et al*., 2023, Preprint). The first copy got lost in the land plant lineage (marked by the black X), while the MIKC-type copy experienced Duplication ② that generated the precursors of the Type I and Type II clade in land plants (Qiu *et al*., 2023). Before the subsequent divergence of the MRCA of land plants, the Type I clade split into M-type and MIKC*-type through Duplication ③ (Qiu *et al*., 2024). The MIKC*-type and MIKC^C^-type precursors each had specific K-box exon duplications (Rümpler *et al*., 2023), while the M-type lost the whole K-box, and experienced Duplication ④ that diverged into the Mα and Mβ clades (Parenicová *et al*., 2003; Qiu and Köhler, 2022). In the MRCA of ferns and seed plants, the MIKC*-type underwent Duplication ⑤ and formed the P-clade and the S-clade (Kwantes *et al*., 2012). In the MRCA of angiosperms, Mγ and Mβ started to differentiate after Duplication ⑥ (Gramzow *et al*., 2014; Qiu and Köhler, 2022), and during the diversification of angiosperms, Mα* TFs specialized from other Mα paralogues after Duplication ⑦ (Qiu and Köhler, 2022). An alternative hypothesis by Han *et al*. (2025) states that Duplication ② took place before the divergence of charophytes (marked by the dashed circle ②), and charophytes only inherited MIKC-type genes orthologous to the Type II genes; however, this scenario is less parsimonious as it requires an assumption of at least five independent gene losses of the Type I lineages across paraphyletic charophytes. The SRF-like genes in core chlorophytes, which putatively originated by horizontal gene transfer (Qiu *et al*., 2023; Han *et al*., 2025), are not shown. Abbreviations labelled for the structures of MADS-box TFs are: MADS (M), Intervening (I), Keratin-like (K), and C-terminal (C) domains.

In addition to the MIKC-type MADS-box genes, a group of MEF2-derived MADS-box genes without the K-box, similar to chlorophytic MADS-box genes, has recently been detected in several charophytes, which likely resemble the ancestral form pre-dating the origin of the K domain (Gramzow *et al*., 2023, Preprint; Rümpler *et al*., 2023; Feng *et al*., 2024; Han *et al*., 2025). These ancestral pre-M-type genes are distinct from the land plant M-type genes and were not inherited by land plants (Gramzow *et al*., 2023, Preprint). Altogether, we summarize the current model on the evolution of MADS-box genes in green plants in Fig. 1. It should be noted that a group of highly divergent MADS-box genes has been detected in some genomes of core chlorophytes that is consistently absent in other sister green algal lineages (Nayar and Thangavel *et al*., 2021; Liang *et al*., 2023; Qiu *et al*., 2023; Han *et al*., 2025). These sequences poorly match either SRF or MEF2 genes, and are only marginally better aligned with fungal SRF genes, while phylogenetic inferences weakly suggest they might cluster with SRF genes. Therefore, horizontal gene transfer of a fungal SRF gene is speculated as the origin of these core chlorophyte-specific SRF-like MADS-box genes (Qiu *et al*., 2023; Han *et al*., 2025).

### M-type MADS-box transcription factors

A pioneering study classified M-type genes into Mα, Mβ, and Mγ subfamilies based on phylogenetic clustering (Parenicová *et al*., 2003). Their typical high copy numbers suggest functional redundancy, therefore higher-order multiple mutations may be necessary to reveal a detectable phenotype (Parenicová *et al*., 2003). In addition, many M-type genes show very low or undetectable expression, also usually restricted to small compartments of flowers and seeds. These features imply that some M-type genes are pseudogenes (Kofuji *et al*., 2003), aligning with the hypothesis that M-type genes undergo a rapid birth-and-death evolutionary dynamic involving frequent gene duplication and pseudogenization (Nam *et al*., 2004). The first functional insight into M-type genes came from studies of *PHERES1* (*PHE1*, also known as *AGL37*), a paternally expressed imprinted gene specifically active in early endosperm development (Köhler *et al*., 2003, 2005). Arabidopsis *PHE1* is epigenetically regulated by Polycomb Repressive Complex 2 (PRC2), where the maternal allele is repressed by PRC2 in the central cell and remains silenced in the endosperm, and misregulation of *PHE1* expression is tightly linked to abnormal endosperm development (Köhler *et al*., 2003, 2005; Batista *et al*., 2019). PHE1 is a key regulator of an endosperm regulatory network, with its targets, such as *HAIKU2*, *YUC10* and *ZHOUPI*, having a variety of functions in endosperm developmental pathways (Batista *et al*., 2019). Therefore, the origin of PHE1, an Mγ-type MADS-box TF, may be closely connected to the evolution of the endosperm.

**Fig. 2.**
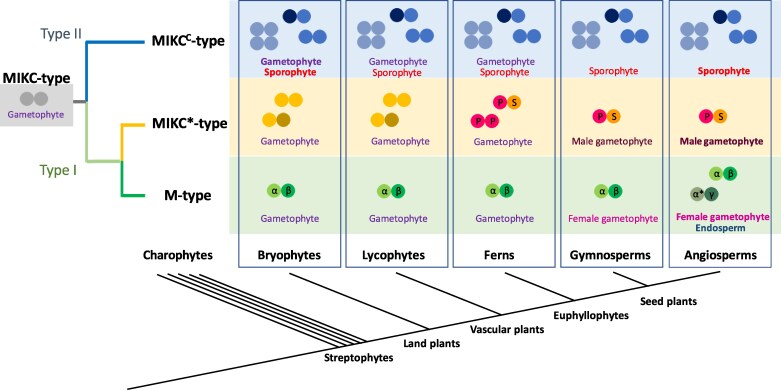
Functional divergence of MADS-box transcription factors (TFs) in land plants. Shown are the generation/tissue type (with lineage-specific or gene-specific exceptions) where each type of MADS-box TFs has major functions. Bold showing functions inferred by functional evidence from mutant studies, otherwise inferred by expression evidence. The MIKC-type TFs in charophytes are expressed exclusively in gametophytes and likely represent the ancestral function of MADS-box TFs in land plants. Type I TFs maintain the functional requirement in gametophytes. In seed plants, MIKC*-type and M-type TFs regulate male and female gametophytes, respectively, and in angiosperms, M-type TFs have neofunctionalized in the endosperm. MIKC^C^-type TFs not only inherited the gametophytic functions but evolved new functions in sporophytes, as seen in non-seed plants. In seed plants, MIKC^C^-type TFs are more pronounced for the diversification of sporophytic development. The MIKC-type TFs in charophytes typically form homodimers but not tetramers, suggesting homodimerization is the ancestral status. M-type TFs majorly form heterodimers between Mα and Mβ/γ TFs, likely across land plants, while the evidence only comes from angiosperm M-type TFs. MIKC*-type TFs can form homodimers and heterodimers before the duplication of P-S clades, after which P-S heterodimers are predominantly formed. MIKC^C^-type TFs can also homodimerize and heterodimerize majorly with other MIKC^C^-type TFs, and additionally they can tetramerize to form the famous floral-quartet-like complexes with a combination of the same one or up to four paralogous TFs (the heterotetramer scenario is not explicitly shown). References for the function and/or expression include but are not limited to: Hasebe *et al*. (1998), Tanabe *et al.* (2005), Zobell *et al*. (2010), Masiero *et al*. (2011), Kwantes *et al*. (2012), Liu *et al*. (2013), Gramzow *et al*. (2014), Theißen *et al*. (2016), Koshimizu *et al*. (2018), Ambrose *et al*. (2021), Qiu and Köhler (2022).

Consistent with this idea, the ancestral Mγ gene can be inferred as an angiosperm-specific duplicate of Mβ genes (Qiu and Köhler, 2022). Mγ-type TFs function as heterodimers with a distinct group of Mα-type TFs, designated Mα*-type, which likewise diverged only in angiosperms. Both Mγ-type and Mα*-type genes have conserved predominant expression in the endosperm across angiosperms, suggesting a shared functional requirement for Mγ-Mα* heterodimers during the evolution of this embryo nourishing tissue. Together, these findings support a model in which duplications within the M-type MADS-box lineage gave rise to angiosperm-specific Mγ and Mα* genes that subsequently co-evolved into key regulators of endosperm development (Figs 1, 2; Qiu and Köhler, 2022).

Supporting this model, an expanding body of work has revealed the regulatory functions for many Mγ-type TFs in endosperm development, in addition to PHE1 and its tandem duplicate PHE2 (Table 1). In Arabidopsis, Mγ-type genes such as *AGL36*, *AGL45*, *AGL80*, and *AGL90*, as well as their orthologues in *Capsella* species, have been implicated in endosperm regulation (Josefsson *et al*., 2006; Portereiko *et al*., 2006; Walia *et al*., 2009; Shirzadi *et al*., 2011; Hoffmann *et al*., 2022; Dziasek *et al*., 2024). Similar functions have been identified for *FveAGL80* in strawberry (Guo *et al*., 2022), *OsMADS82*, *OsMADS87*, *OsMADS88*, and *OsMADS89* in rice (Folsom *et al*., 2014; Chen *et al*., 2016; Paul *et al*., 2020), and for *TaMADS-GS-A* and *TaMADS-GS-D* in wheat (J. Zhang *et al*., 2024). As interaction partners of Mγ-type TFs, several Mα*-type TFs also play important roles in endosperm development, including AGL28, AGL40, and AGL62 in Arabidopsis and their *Capsella* orthologues (Kang *et al*., 2008; Walia *et al*., 2009; Roszak *et al*., 2011; Hehenberger *et al*., 2012; Kradolfer *et al*., 2013; Figueiredo *et al*., 2015, 2016; Kirkbride *et al*., 2019; Hoffmann *et al*., 2022; Dziasek *et al*., 2024), FveAGL62 in strawberry (Guo *et al*., 2022), and OsMADS78 and OsMADS79 in rice (Paul *et al*., 2020). Knock-out or overexpression of these Mγ- and Mα*-type genes typically results in aberrant endosperm development and reduced seed viability, while embryogenesis itself is usually unaffected.

**Table 1.** Functional studies on M-type MADS-box genes

Function summary	Species	Subfamily	Gene	Functional roles	Reference
Regulators of central cell differentiation network in the female gametophyte	*Arabidopsis thaliana*	Mγ	*AGL80*	AGL80 is required for central cell differentiation.	Portereiko *et al*. (2006)
				AGL80 is required for the expression of *DEMETER* and *DD46* in the central cell.	
				AGL80 specifies central cell fate by suppressing the expression of accessory cell-specific genes in the central cell.	Zhang *et al*. (2020)
		Mα*	*AGL61*	AGL61 is required for central cell differentiation.	Bemer *et al*. (2008)
				AGL61 specifies central cell fate by suppressing the expression of accessory cell-specific genes in the central cell.	Steffen *et al*. (2008)
Regulators of endosperm development network during the nuclear phase	*Arabidopsis* species	Mγ	*PHE1*/*AGL37*	Increased *PHE1* expression contributes to endosperm overproliferation in *prc2* mutant seeds.	(Köhler *et al*., 2003; 2005)
				Up-regulation of *PHE1* plays a causal role in hybrid seed failure between *A. thaliana* and *A. arenosa*.	Josefsson *et al*. (2006)
				PHE1 directly targets imprinted genes and non-imprinted key regulators of endosperm development.	Batista *et al*. (2019)
				PHE1 activates endosperm-specific invertase inhibitors *InvINH1* and *InvINH2*, impacting on embryo growth rate.	Hoffmann *et al*. (2022)
			*AGL80*	AGL80 is required for endosperm initiation.	Portereiko *et al*. (2006)
			*AGL90*	Up-regulation of *AGL90* plays a causal role in the endosperm-based postzygotic barrier between *A. thaliana* and *A. arenosa*.	Walia *et al*. (2009)
				AGL90 activates endosperm-specific invertase inhibitors InvINH1 and InvINH2, impacting on embryo growth rate.	Hoffmann *et al*.(2022)
			*AGL45*	AGL45 activates endosperm-specific invertase inhibitors *InvINH1* and *InvINH2*, impacting on embryo growth rate.	Hoffmann *et al*. (2022)
		Mα*	*AGL62*	AGL62 plays a causal role in the endosperm-based postzygotic barrier between *A. thaliana* and *A. arenosa*.	Walia *et al*. (2009)
				AGL62 is required for endosperm proliferation and premature cellularization.	Kang *et al*. (2008)
				AGL62 produces a signal in the endosperm that initiates seed coat development.	Roszak *et al*. (2011)
				AGL62 plays a causal role in establishing seed defects in *prc2* mutants and interploidy crosses.	Hehenberger *et al*. (2012)
				AGL62 is a dosage-sensitive regulator of seed size.	Kradolfer *et al*. (2013)
				AGL62 is required for auxin-dependent endosperm proliferation.	Figueiredo *et al*. (2015)
				AGL62 regulates auxin transport from the endosperm to the integuments to initiate seed coat development.	Figueiredo *et al*. (2016)
				AGL62 activates endosperm-specific invertase inhibitors *InvINH1* and *InvINH2*, impacting on embryo growth rate.	Hoffmann *et al*. (2022)
			*AGL40*	AGL40 regulates seed size and weight.	Kirkbride *et al*. (2019)
				AGL40 activates endosperm-specific invertase inhibitors *InvINH1* and *InvINH2*, impacting on embryo growth rate.	Hoffmann *et al*. (2022)
		Mα	*AGL91*	AGL91 regulates seed size and weight.	Kirkbride *et al*. (2019)
	*Capsella* species	Mγ	*PHE2*/*AGL38*	AGLs activate genes associated with interspecific hybrid barrier across *Capsella* species.	Dziasek *et al*. (2024)
				*AGL*s are *trans*-targets of endosperm-specific siRNAs regulating interspecific hybrid barriers across *Capsella* species.	
		Mα*	*AGL62*	AGLs activate genes associated with interspecific hybrid barriers across *Capsella* species.	
				*AGL*s are *trans*-targets of endosperm-specific siRNAs regulating interspecific hybrid barriers across *Capsella* species.	
			*AGL28*	AGLs activate genes associated with interspecific hybrid barriers across *Capsella* species.	
				*AGL*s are *trans*-targets of endosperm-specific siRNAs regulating interspecific hybrid barriers across *Capsella* species.	
	*Fragaria vesca*	Mγ	*FveAGL80*	FveAGL80 activates auxin and gibberellic acid synthesis in the endosperm.	Guo *et al*. (2022)
		Mα*	*FveAGL62*	FveAGL62 activates auxin and gibberellic acid synthesis in the endosperm.	
	*Oryza sativa*	Mγ	*OsMADS87*	*OsMADS87* delays endosperm cellularization.	Folsom *et al*. (2014)
			*OsMADS89*	OsMADS89 regulates endosperm cellularization and seed size.	Chen *et al*. (2016)
				OsMADS89 is required for the nuclear localization of the Mα*-Mγ heterodimers.	Paul *et al*. (2020)
			*OsMADS82*	*OsMADS82* delays endosperm cellularization.	Folsom *et al*. (2014)
				OsMADS82 responds to heat stress and regulates endosperm cellularization and seed size.	Chen *et al*. (2016)
			*OsMADS88*	OsMADS88 responds to heat stress and regulate cellularization and seed size.	Chen *et al*. (2016)
		Mα*	*OsMADS78*	*OsMADS78* delays endosperm cellularization.	Paul *et al*. (2020)
				OsMADS78 regulates genes involved in auxin transport and signalling and starch biosynthesis.	
			*OsMADS79*	*OsMADS79* delays endosperm cellularization.	
				OsMADS79 regulates genes involved in auxin transport and signalling and starch biosynthesis.	
	*Triticum aestivum*	Mγ	*TaMADS-GS*	*Tamads-gs* knockout lines have delayed endosperm cellularization.	J. Zhang *et al.* (2024)
				*Tamads-gs* knockout lines have fewer endosperm nuclei and cells, smaller grain size and lower grain weight.	
				TaMADS-GS regulates the cytokinin pathway by repressing cytokinin oxidase/dehydrogenases (*CKXs*) genes.	

Endosperm development follows three major modes: nuclear, cellular, and the less common helobial type. In the nuclear type, nuclei initially undergo repeated divisions without cell wall formation, resulting in a coenocyte; cellularization occurs later and is vital for seed survival. In the cellular type, cytokinesis accompanies each nuclear division. The cellular type is inferred as ancestral, represented in most *Amborellales*, *Nymphaeales*, and *Austrobaileyales* (ANA-grade), and magnoliid species, whereas in monocots and eudicots there have been independent transitions to the nuclear type (Friedman, 1994; Geeta, 2003). All species with functionally characterized M-type TFs discussed above possess nuclear-type endosperm. Many of these Mγ-type and Mα*-type genes show expression specifically during the nuclear phase and become down-regulated or silenced after cellularization. Their expression levels positively correlate with the extent of free nuclear proliferation. Knockout mutants typically undergo premature cellularization, producing smaller seeds, while overexpression often delays cellularization, frequently connected with seed lethality due to failure of proper cellularization. One notable exception are the wheat TaMADS-GS homeologs. *Tamads-gs* knockouts have smaller grains with fewer endosperm cells, consistent with reduced proliferation; however, cellularization is paradoxically delayed (J. Zhang *et al*., 2024). This exception suggests that Mγ- and Mα*-type TFs may directly regulate nuclear and/or cell division while only indirectly influencing the timing of cellularization, with lineage-specific variation in how these regulatory pathways crosstalk. Beyond functional studies, numerous Mγ-type and Mα*-type genes are consistently detected in published endosperm and seed transcriptomes (Qiu and Köhler, 2022), supporting the idea that Mγ-Mα* heterodimers play a conserved role in the endosperm regulatory network. Transcriptomic analyses have also revealed that some M-type MADS-box genes are expressed during the cellularized stage of nuclear-type endosperms as well as during the early-stage endosperm of species with cellular-type endosperm (Flores-Vergara *et al*., 2020; Qiu and Köhler, 2022), although these genes have not yet been functionally characterized through mutant analyses. Additionally, in endosperms exhibiting abnormal developmental patterns, which are common phenotypes arising from interspecific or interploidy crosses, many Mγ-type and Mα-type genes are among the most strongly deregulated genes. Such deregulation during defective endosperm development often leads to altered seed phenotypes, proposing that M-type genes are critical regulators of endosperm-based reproductive barriers (Walia *et al*., 2009; Tiwari *et al*., 2010; Lu *et al*., 2012; Rebernig *et al*., 2015; Roth *et al*., 2018; Kirkbride *et al*., 2019; Florez-Rueda *et al*., 2021; Bente and Köhler, 2024; Dziasek *et al*., 2024). Together, these findings further underscore the central regulatory importance of M-type MADS-box genes in endosperm development.

The known functions of M-type MADS-box genes are not limited to the endosperm, but also the precursor of the endosperm, the central cell. In Arabidopsis another Mγ-Mα* heterodimer, AGL80-AGL61(DIANA/DIA), is required for central cell differentiation during development of the female gametophyte (Portereiko *et al*., 2006; Bemer *et al*., 2008; Steffen *et al*., 2008; Zhang *et al*., 2020). While *AGL61* is nearly exclusively expressed in the central cell, occasionally in the very early stage of the endosperm development in the first two to eight nuclei (Bemer *et al*., 2008; Steffen *et al*., 2008), *AGL80* continues expression in early stages of the nuclear endosperm formation (Portereiko *et al*., 2006), suggesting dual functions of some M-type MADS-box genes in both shared and distinct pathways in the female gametophyte and the endosperm.

In addition to the functions in the female gametophyte and the endosperm, some M-type MADS-box genes were reported to be expressed in sporophytic vegetative tissues and in pollen (Parenicová *et al*., 2003; Bemer *et al*., 2010). However, these reported expression patterns are frequently not consistent in different datasets and most of them do not have an evident function in these tissue types. For example, AGL28 was proposed to regulate flowering time, based on the observation that the overexpression of *AGL28* can promote flowering (Yoo *et al*., 2006). However, since *agl28* shows no obvious aberrant phenotype regarding flowering time, it requires further evidence to conclude whether AGL28 has redundant function in flowering regulation as assumed (Yoo *et al*., 2006). Overall, it remains open whether M-type MADS-box genes have an important function in sporophytes and male gametophytes in angiosperms.

At present, nearly all M-type MADS-box TFs with experimentally characterized functions belong to the Mγ-type group and their interacting Mα*-type partners. The only exception is AGL91 in Arabidopsis, which has been identified as a positive regulator of seed size, similar to its paralogous Mα*-type TFs (Kirkbride *et al*., 2019). Little is currently known about the functions of Mβ-type or other Mα-type TFs. A few of these genes show weak expression in the female gametophyte of Arabidopsis, suggesting that they may form functional Mβ-Mα heterodimers involved in female gametophyte development (Bemer *et al*., 2010). Further inspired by evidence from gymnosperm transcriptomes, it is tempting to speculate that Mβ-Mα heterodimers function in the developing gymnosperm female gametophyte, which provides embryo nourishment in a manner analogous to the angiosperm endosperm (Baroux and Grossniklaus, 2019). Based on this idea, while Mβ-type TFs maintain ancestral regulatory roles in maternal tissues, the duplication events that produced the angiosperm-specific Mγ and Mα* clades allowed Mγ-type TFs to undergo neofunctionalization and establish the endosperm regulatory programme (Fig. 2; Qiu and Köhler, 2022). Future functional studies will allow to test this hypothesis and to clarify how Mβ-type TFs and their interacting Mα-type partners operate in angiosperms and gymnosperms. It will also be important to investigate the functions of M-type MADS-box genes in other land plant lineages. Such work may reveal whether their roles in gametophyte development are conserved and may uncover previously unrecognized functions in sporophytes.

### MIKC*-type MADS-box transcription factors

As outlined above, MIKC*-type genes form a clade distinct from all MIKC^C^-type subfamilies; despite containing a K-box, they belong to the Type I clade due to their closer relationship with M-type genes (Qiu *et al*., 2024).

Compared with MIKC^C^-type genes, MIKC*-type genes are less diversified in general. Like other MADS-box TFs, MIKC*-type TFs form dimers to bind DNA and regulate target genes. In the liverwort *Marchantia polymorpha*, the sole MIKC*-type TF, MpMADS1, forms homodimers (Zobell *et al*., 2010). In mosses and lycophytes, lineage-specific duplicates exhibit both homo- and heterodimerization, for example, in *Selaginella moellendorffii*, SmMADS2 and SmMADS10 form homodimers, while SmMADS4 and SmMADS10 form heterodimers (Kwantes *et al*., 2012). In euphyllophytes, which are plants with true leaves, including ferns and seed plants, MIKC*-type genes fall into two subclades, the P-clade and the S-clade, originating from a duplication after the divergence of lycophytes from the rest of vascular plants (Fig. 1; Kwantes *et al*., 2012; Liu *et al*., 2013). Subsequent divergence shaped an obligate heterodimerization system between P- and S-clade proteins (Fig. 2). In the fern *Ceratopteris richardii*, S-clade TFs CRM14 and CRM15 heterodimerize exclusively with P-clade TFs CRM13 and CRM16, while P-clade TFs retain limited homodimerization capacity (Kwantes *et al*., 2012). This trend to heterodimerization is strongest in angiosperms, where P-S heterodimerization appears obligatory, as demonstrated in Arabidopsis and rice (Verelst *et al*., 2007a; Liu *et al*., 2013). Notably, in California poppy *Eschscholzia californica*, the S-clade TF EcMADS2 can homodimerize, whereas the P-clade EcMADS1 does not (Kwantes *et al*., 2012), indicating that the transition to obligate heterodimerization was not uniform. The loss of homodimerization potentially drove the retention of both clades. Co-expression of P- and S-clade genes ensures the formation of functional heterodimers in confined tissues. In Arabidopsis and rice, both clades are predominantly expressed in the male gametophyte, pollen (Verelst *et al*., 2007a, b; Adamczyk and Fernandez, 2009; Liu *et al*., 2013). The poppy gene *EsMADS1* is expressed at low levels in sporophytic tissues, while *EsMADS2* is strongly enriched in pollen where the function regulated by the P-S heterodimer is likely restricted (Kwantes *et al*., 2012). In ferns, MIKC*-type genes are expressed in male or hermaphrodite gametophytes as well as some sporophytic tissues, such as the root (Kwantes *et al*., 2012). For the orthologues diverged before the P-S duplication, for instance, in the lycophytes, *Selaginella moellendorffii* and *Selaginella pallescens*, two of the three MIKC*-type genes are specifically expressed in microsporangia, which produce microspores (Kwantes *et al*., 2012), and *LAMB1* from *Lycopodium annotinum* is specifically expressed in developing strobili containing sporogeneous cells (Svensson *et al*., 2000). These data suggest that the ancestral role of MIKC*-type genes is connected to microspore and male gametophyte development. Additional evidence comes from 11 MIKC*-type genes in the moss *Funaria hygrometrica*, and two *Physcomitrium* MIKC*-type genes, which are preferentially expressed in the gametophytes (Riese *et al*., 2005; Zobell *et al*., 2010). Remarkably, the single-copy MpMADS1 can rescue loss of P-S heterodimers in Arabidopsis (Zobell *et al*., 2010). These findings suggest a conserved function of MIKC*-type MADS-box genes in land plant male gametophytes (Fig. 2).

Functional characterizations of MIKC*-type genes initially focused on Arabidopsis. There are three P-clade genes (*AGL30*, *AGL65*, and *AGL94*) and two S-clade genes (*AGL66* and *AGL104*) expressed in Arabidopsis pollen that can form five P-S heterodimers, AGL30-AGL66, AGL30-AGL104, AGL65-AGL66, AGL65-AGL104, and AGL94-AGL66. Mutant analyses revealed high redundancy within clades and between P-S complexes, but complete loss of MIKC* function results in pollen germination and pollen tube growth defects (Verelst *et al*., 2007a, b; Adamczyk and Fernandez, 2009). In rice, the sole P-clade gene *OsMADS68* is required for pollen development, and double mutants of the S-clade genes *OsMADS62* and *OsMADS63* exhibit severe pollen abnormalities, with most pollen arrested at the bicellular stage (Liu *et al*., 2013). These phenotypes are more pronounced than in Arabidopsis, likely reflecting less redundancy. Across both species, MIKC*-type genes are expressed from the unicellular microspore stage through pollen tube growth (Verelst *et al*., 2007a, b; Wang *et al*., 2008; Liu *et al*., 2013), suggesting functional roles throughout male gametophyte development (Fig. 2). Interestingly, the S-clade gene *AGL67*, a Brassicaceae-specific tandem duplicate of *AGL66*, is not expressed in pollen. Instead, it modulates desiccation tolerance during seed maturation and regulates seed dormancy in response to temperature stimuli (González-Morales *et al*., 2016; Narro-Diego *et al*., 2017; Li *et al*., 2020). The functional divergence observed between the recent duplicates of *AGL67* and *AGL66* may have arisen by neofunctionalization of *AGL67*, while subfunctionalization remains an alternative explanation. Extending functional analyses to additional land plant lineages, guided by robust data on their expression and dimerization, will determine how deeply conserved MIKC*-type roles are in male gametophyte development and whether they also contribute to sporophyte development.

### The regulatory network governed by type I MADS-box transcription factors

The reclassification of MIKC*-type MADS-box TFs into the gametophytic Type I clade raises the question of how the sister M-type and MIKC*-type TFs have functionally diverged. The functional evolution of MADS-box TFs concerns not only the TF genes themselves, but also the rewired downstream gene regulatory networks. Type II MADS-box TFs recognize the canonical CArG-box, CC(A/T)_6_GG, and variants such as the N10-type, C(A/T)_8_G (Aerts *et al*., 2018). Arabidopsis PHERES1 is currently the only plant Type I TF with known genome-wide DNA-binding sites *in vivo*, and the binding motifs are predominantly the canonical CC(A/T)_6_GG motif and a derived TTTCC(A/T)_5_ form (Batista *et al*., 2019). As *PHE1* expression gradually declines during seed development, most of its target genes are correspondingly down-regulated, with roughly one quarter showing a significant positive correlation with *PHE1* transcript levels. Notably, *PHE1* overexpression leads to significant up-regulation of more than 30% of its target genes, whereas a subset of targets is instead up-regulated in the *phe1 phe2* double mutant. Together, these observations indicate that PHE1 functions primarily as a transcriptional activator, although it can act as a repressor in certain contexts (Batista *et al*., 2019). PHE1 targets are enriched in pathways related to auxin and brassinosteroid synthesis and signalling, diverse metabolic processes, and regulatory modules controlling seed growth. Many TF families are over-represented among these targets, placing PHE1 high up in the regulatory hierarchy. Importantly, several other M-type MADS-box genes, and *PHE1* itself, contain PHE1-binding motifs, suggesting extensive auto- and cross-regulatory interactions within this family and synergistically enabling rapid amplification of functional M-type heterodimer dosage (Batista *et al*., 2019). The regulatory landscape of M-type MADS-box TFs has been dynamically reshaped through the exaptation of transposable elements. A notable example is a group of DNA transposons, helitrons, which distributed CArG-box motifs throughout the genome. Some of these motifs were subsequently domesticated by PHE1, thereby expanding and rewiring its regulatory network by recruiting nearby genes (Batista *et al*., 2019). In sporophytic tissues, helitron-derived CArG-boxes are typically masked by DNA methylation, but they become exposed in the endosperm due to global hypomethylation, enabling PHE1 binding while preventing access by sporophytic MIKC^C^-type TFs (Qiu and Köhler, 2020).

Direct targets of MIKC*-type TFs, and the *cis*-regulatory features they recognize, remain less comprehensively studied with genomic approaches. Microarray-based transcriptome profiling of MIKC*-type mutants in Arabidopsis identified about 200 putative direct targets of MIKC*-type TFs (Verelst *et al*., 2007a, b). *In silico* motif enrichment and *in vitro* binding assays indicated that these TFs preferentially bind an N10-type motif, C(A/T)_8_G. Thus, differentiation in target site specificity may underlie the regulatory divergence between MIKC*-type and M-type MADS-box TFs. Because MIKC*-type gene expression increases towards the late stages of pollen development, many of their candidate targets show parallel up-regulation during pollen maturation and reduced expression in mikc* mutants, consistent with a role for MIKC*-type TFs as transcriptional activators. However, a subset of targets exhibits the opposite pattern, indicating that MIKC*-type TFs can also function as repressors (Verelst *et al*., 2007a, b; Adamczyk and Fernandez, 2009). Several targets are known regulators of pollen development and germination, for example, JINGUBANG, which inhibits pollen germination by regulating the jasmonic acid biosynthesis pathway (L. Zhang *et al*., 2024), as well as BUPS1 and LRX9, which interact with RALF peptides to promote pollen tube growth (Ge *et al*., 2017; Mecchia *et al*., 2017; Zhu *et al*., 2018). As with PHE1, many transcription factor families are represented among the putative MIKC*-type targets, including two MIKC*-type genes, *AGL30* and *AGL65*. The preferential recognition of the N10-type motif is conserved in rice MIKC*-type TFs and also in the *Marchantia* MIKC*-type homodimer MpMADS1 (Zobell *et al*., 2010; Liu *et al*., 2013), revealing deep evolutionary conservation of the binding site of MIKC*-type transcription factors. Future comprehensive genomic identification of direct MIKC*-type targets across additional lineages can help understand how MIKC*-type TFs generated such diverse regulatory outcomes in the male gametophytes of land plants while maintaining a largely invariant mode of DNA interaction.

Beyond their distinct DNA-binding preferences, MIKC*-type MADS-box genes in angiosperms also show expression patterns that are almost mutually exclusive with those of M-type genes, with the former restricted largely to pollen and the latter predominantly active in the central cell and its descendent endosperm. However, a small subset of predicted MIKC*-type targets (Verelst *et al*., 2007b) also appear among PHE1 target genes in the endosperm (Batista *et al*., 2019). These shared targets point to conserved regulatory roles for MADS-box TFs in both pollen and endosperm, including control of the cell cycle (CDK8/CDKE1), abscisic acid biosynthesis and signalling (NCED6), chromatin and epigenetic regulation (VEL1), and cytoskeletal organization (TUB4). Interestingly, two MIKC*-type target genes encoding invertase inhibitors are not expressed in the endosperm and are not bound by PHE1. However, their paralogues, InvINH1 and InvINH2, which are endosperm specific, are regulated by M-type TFs (Hoffmann *et al*., 2022). This highlights how the same biological pathway can be partitioned and differentially controlled by MIKC*-type versus M-type TFs through subfunctionalization.

### Divergence in dimerization and protein–protein interactions

Another major axis of MADS-box transcription factor evolution is the diversification of dimerization patterns. The functional unit required for DNA binding and dimerization comprises two α-helices joined by a pair of antiparallel β-strands. However, the canonical definition of the MADS domain historically included only the first helix and the β-strands. The second helix was long thought to derive from different domains in different lineages: the SAM domain in animal and fungal SRF proteins, the MEF2 domain in animal and fungal MEF2 proteins, and the I domain in plant MIKC^C^-type and MIKC*-type TFs (Riechmann *et al*., 1996; Lai *et al*., 2021). Recent analyses have also identified an I-like region in plant M-type TFs that forms a true second helix (Lai *et al*., 2021; Qiu *et al*., 2023). These findings demonstrate that all MADS-box TFs share a homologous second helix that is both structurally and functionally conserved. Consequently, an expanded definition of the MADS domain has been proposed to encompass this full helix-strand-helix module, which likely evolved as a single functional unit (Qiu *et al*., 2023).

Within this framework, SRF-type TFs represent a notable departure, as their second helix diverged from the ancestral MEF2-type configuration, turning into an opposite orientation and acquiring a distinctive kink. As a result, SRF-type proteins cannot dimerize with MEF2-type TFs, restricting species with one SRF and/or one MEF2 TF to only homodimers (Shore and Sharrocks, 1995). When either TF is duplicated, additional heterodimers become possible, as illustrated by MCM1-ARG80 interaction in yeast and combinatorial MEF2 dimerization in mammals (Martin *et al*., 1994; Jamai *et al*., 2002; Aude-Garcia *et al*., 2010). In land plants, the expansion of MADS-box families dramatically multiplied these combinatorial possibilities (de Folter *et al*., 2005). Partner-specific dimerization allows a single MADS-box TF to access distinct sets of target genes (Lai *et al*., 2021; van Mourik *et al*., 2023), while extensive diversification in C-terminal regions further enriches protein–protein interaction potentials and the assembly of regulatory complexes. These mechanisms enabled far-reaching divergence of regulatory programmes without major innovation in DNA-binding sequence specificity.

To date, there is little evidence for extensive functional interaction between the three major plant MADS-box groups. The evolution of the dimerization patterns between the three major types of land plant MADS-box TFs can be contextualized by examining charophytic MADS-box TFs, which likely pre-date the Type I and Type II split and remain low in copy number (Qiu *et al*., 2023). Charophytic MADS-box TFs, especially single-copy ones, readily form homodimers but typically lack the ability to tetramerize, suggesting that homodimerization reflects the ancestral state of land plant MADS-box TFs (Fig. 2; Rümpler *et al*., 2023). After the first duplication split off the MIKC^C^-type lineage and a combined MIKC*-type and M-type lineage, the ancestral MIKC^C^-type TF retained the ability to homodimerize. Following further duplications, many MIKC^C^-type TFs are capable of both homo- and heterodimerization mainly with other MIKC^C^-type, and furthermore, can assemble into higher-order tetramers mediated by the K domain, greatly expanding their combinatorial and functional diversity (Fig. 2; Smaczniak *et al*., 2012; Theißen *et al*., 2016). The sister lineage diverged further into MIKC*-type and M-type TFs, with the latter splitting into the obligately interacting Mα and Mβ/γ subtypes (Fig. 2; Qiu and Köhler, 2022). MIKC*-type TFs retained facultative homodimerization until the emergence of euphyllophytes, where duplication into P- and S-clades generated paralogues that gradually evolved strict P-S heterodimerization (Fig. 2). Despite having a K domain, MIKC*-type TFs are likely not able to form tetramers because of different exon duplications in the coding K-box region compared to the MIKC^C^-type TFs (Rümpler *et al*., 2023).

### Functional evolution of MADS-box TFs for terrestrialization

**Fig. 3.**
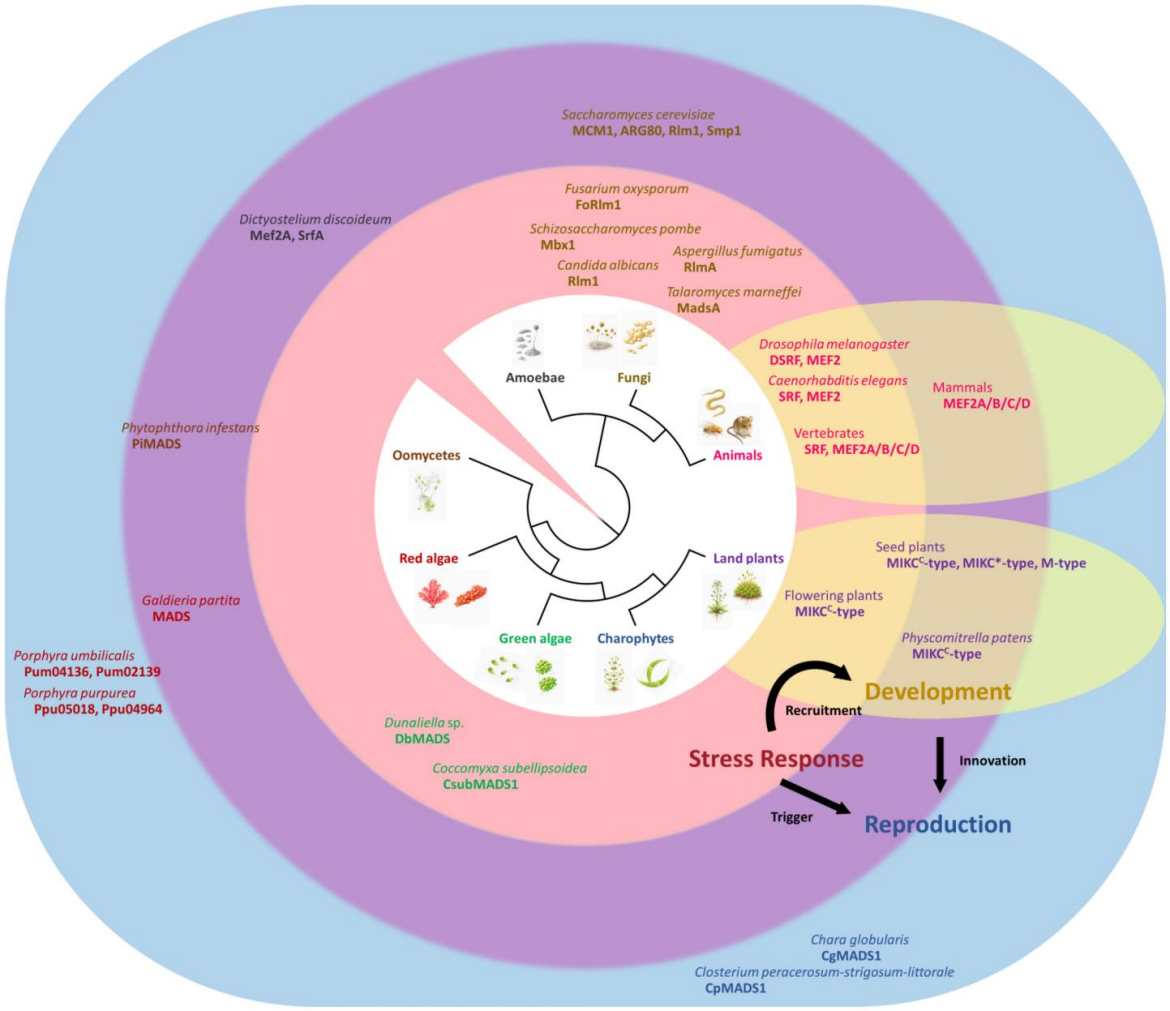
Conserved and derived major functional themes of MADS-box transcription factors (TFs) across eukaryotes. Genes with experimentally evident functions locate in the correspondingly coloured functional zones: red, stress response; blue, reproduction; purple, intersection between stress response and reproduction; yellow, development and body planning for the two main lineages that convergently evolved multicellularity, animals and land plants. Species and genes have the same colours as the aligned taxonomic groups in the central phylogeny to which they belong. The eukaryotic phylogeny follows the topology in Burki *et al*. (2020). The ancestral function of MADS-box TFs can be inferred to be stress response, which is inherited in many extant MADS-box TFs across eukaryotes. One typical type of stress response is the transition into reproduction, when MADS-box TFs are key regulators of such reprogramming under certain environmental or cellular cues. In animals and land plants, stress-responsive MADS-box TFs have been independently recruited into developmental programmes, which integrate environmental and intrinsic signals to architect diverse body patterns that are adaptive to terrestrialization, including developmental innovations in reproductive structures and behaviours.

Following the ancient duplication of the Type I and Type II lineages, the MADS-box gene family expanded dramatically in land plants, providing the genetic material for extensive functional diversification. This expansion underlies the increasing developmental complexity seen across extant plant groups and likely contributed to their successful adaptation during terrestrialization (Theißen *et al*., 1996; Qiu *et al*., 2024). In striking contrast, most other eukaryotes possess only a handful of MADS-box genes (Theißen *et al*., 1996; Thangavel and Nayar, 2018; Qiu *et al*., 2023). To infer the ancestral functions from which the plant repertoire evolved, it is therefore informative to examine the functions of MADS-box genes in unicellular or minimally differentiated multicellular lineages, where the gene family has remained at low copy number (Fig. 3).

#### A conserved regulatory link between stress response and reproduction

At the cellular level, many MADS-box TFs in animals, yeasts, and plants have been shown to directly regulate the cell cycle, determining cell division or differentiation (Messenguy and Dubois, 2003; Batista *et al*., 2019). Such cellular function can be tightly connected to stress responses with evidence from MADS-box genes studied in a diverse array of eukaryotes (Messenguy and Dubois, 2003; Castelán-Muñoz *et al*., 2019; Qiu *et al*., 2023). Across fungi, species typically have only one or two MEF2-type and one or two SRF-type MADS-box genes. In budding yeast, the MEF2-type TFs Rlm1 and Smp1 are required for cell wall integrity and stress resistance (Dodou and Treisman, 1997; de Nadal *et al*., 2003; Staleva *et al*., 2004). Analogous roles are observed for the fission yeast homologue Mbx1 and for Rlm1 homologues in the human pathogen *Candida albicans* (Takada *et al*., 2010; Heredia *et al*., 2020). Similarly, stress-responsive functions are conserved in filamentous fungi, and loss of MEF2-type TFs compromises stress tolerance and reduces virulence, including FoRlm1 in the plant pathogen *Fusarium oxysporum* (Ding *et al*., 2020), and for RlmA as well as MadsA in the human pathogens *Aspergillus fumigatus* (Rocha *et al*., 2016) and *Talaromyces marneffei* (Wang *et al*., 2018), respectively. SRF-type TFs, such as yeast MCM1 and ARG80, also play central roles in modulating gene expression in response to environmental cues (Messenguy and Dubois, 2003). The consistent association of MADS-box TFs with stress-responsive regulation indicates that SRF and MEF2 likely originated from a single ancestral stress-responsive TF (Fig. 3). After gene duplication, they were probably only weakly differentiated and partially redundant, regulating overlapping sets of genes. This redundancy would have allowed one paralogue to compensate for the loss of the other. Under this scenario, the ancestral MEF2-type gene in the unicellular ancestor of the plant lineage may have functionally replaced SRF following its loss.

In many unicellular or simple multicellular organisms, sexual or asexual reproduction is triggered by environmental stress, and the resulting dormant spores or zygotes often show enhanced stress tolerance (Bernstein and Bernstein, 2010; Wallen and Perlin, 2018). It is therefore plausible that the ancestral stress-response role of MADS-box TFs was repeatedly co-opted into reproductive development (Fig. 3). In line with this hypothesis, in budding yeast Rlm1 promotes sporulation, the starvation-induced gametogenesis, by regulating early meiotic gene expression (Piccirillo *et al*., 2015), while MCM1 and ARG80 also function in the mating pheromone pathway (Messenguy and Dubois, 2003). Evidence of this ancient functional linkage of MADS-box TFs between stress and reproduction extends into the social amoeba *Dictyostelium discoideum*, which Mef2A promotes fruiting body formation and spore production under starvation, while SrfA is required for spore differentiation in response to extracellular signals (Escalante *et al*., 2003; Galardi-Castilla *et al*., 2013). Similarly, in the oomycete pathogen *Phytophthora infestans*, the single-copy MEF2-type TF PiMADS is strongly induced during asexual sporulation and regulates most sporulation-associated genes (Leesutthiphonchai and Judelson, 2018).

A similar pattern is evident in the green lineage (Fig. 3). In microalgae, CsubMADS1 from *Coccomyxa subellipsoidea* plays a central role in stress tolerance (Nayar and Thangavel, 2021). *CsubMADS1* is most highly expressed during the lag phase, and its overexpression causes growth retardation and the formation of polyploid giant cells, consistent with a cell-cycle regulatory function in resilience and acclimation (Nayar and Thangavel, 2021). Similarly, *DbMADS* from *Dunaliella* sp. FACHB-847 is also highly expressed during the lag phase and represses carotenoid biosynthesis while the accumulation of these pigments is tightly linked to environmental stresses (Liang *et al*., 2023). Additional support for conserved stress-reproduction linkages comes from red algae. The single-copy MADS-box TF in unicellular *Galdieria* regulates the transition from the cell wall-less haploid phase to the cell-walled diploid phase under acetic acid stress (Hirooka *et al*., 2022). In *Porphyra umbilicalis*, a MEF2-type TF is up-regulated in asexual spores relative to the vegetative blade, while in *Porphyra purpurea*, a related *MEF2* gene shows higher expression in sporophytic filaments than in the gametophytic blade (Stiller *et al*., 2012). These patterns imply that MEF2 TFs in red algae contribute to both sexual and asexual reproduction and may have acquired specific roles during generation alteration between sporophytes and gametophytes. In charophytes, the successive sister groups of land plants, the link to reproductive differentiation becomes even clearer. In *Chara globularis*, a MIKC-type TF is specifically expressed in the oogonium and antheridium and remains enriched in the differentiating egg and spermatozoid until zygote formation (Tanabe *et al*., 2005). Moreover, evidence comes from a class of unicellular and filamentous charophytes, Zygnematophyceae, the closest relatives of land plants (Hess *et al*., 2022). It is in the *Closterium peracerosum-strigosum-littorale* complex that the MIKC-type gene is precisely up-regulated at the onset of gametangial differentiation and declines after fertilization (Tanabe *et al*., 2005). Because charophytes have a gametophyte-dominant life cycle in which the zygote is the only diploid stage, these expression profiles indicate that the immediate precursors of land-plant MADS-box genes had already acquired roles in controlling gamete differentiation. As land plants transitioned to the terrestrial environment, which was far more variable and stressful than their ancestral marine habitats, these ancestral regulatory roles were expanded. Beyond the best-known functions in floral patterning and flowering time regulation in sporophytes, MADS-box genes regulate the pre-zygotic gametophyte development and post-zygotic embryo nourishment, and they also retain important roles in environmental response across land plants (Castelán-Muñoz *et al*., 2019). This functional spectrum is consistent with the hypothesis of a deep evolutionary origin of stress-responsive regulation and subsequent recruitment in architecting reproductive programmes (Fig. 3).

#### Divergence of MEF2-type MADS-box transcription factors contributed to multicellularity

As the metazoan lineage evolved multicellularity, *SRF* and *MEF2* genes, although far fewer in number than their plant counterparts, also evolved central roles in developmental regulation (Fig. 3). Both SRF and MEF2 function in embryonic patterning and continue to regulate muscle development throughout adulthood. As in plants, the transition to terrestrial environments benefited from the ancestral stress-responsive functions of MADS-box genes. These roles were integrated into the emerging developmental networks of animals. In *Drosophila melanogaster*, MEF2 participates in pathways controlling oxidative stress, circadian rhythms, and lifespan (Blanchard *et al*., 2010; Vrailas-Mortimer *et al*., 2011). In *Caenorhabditis elegans*, MEF2 promotes entry into the dauer stage, a stress-resistant larval form, by regulating chemoreceptor genes (van der Linden *et al*., 2007). In vertebrates, MEF2 TFs respond to oxidative stress and fluid shear stress to regulate cardiac muscle growth, and they become activated at injury sites to promote vascular remodelling (Potthoff and Olson, 2007). Across metazoans, therefore, MEF2 TFs link deeply conserved stress responses to tissue differentiation and body patterning. The adoption of stress-responsive transcription factors into developmental programmes likely conferred major advantages in multicellular organisms by allowing growth, differentiation, and patterning to be coordinated with extrinsic environmental fluctuations. Specifically, *MEF2* genes are expressed predominantly in the early mesoderm, the precursor of muscle, vasculature, and much of the nervous system, thereby supporting the mobility, structural integrity, and sensory capacity, adaptive characteristics of metazoans to manage the increasing environmental exposure and complexity associated with life on land (Potthoff and Olson, 2007). While most classical MEF2 studies were muscle focused, upcoming studies have shown mammalian MEF2 TFs directly regulate a series of genes with important functions in testis, which clearly establish their roles in reproductive function (Daems *et al*., 2014; Di-Luoffo *et al*., 2015).

In vertebrates, the MEF2 subfamily underwent a characteristic expansion and diversification into MEF2A, MEF2B, MEF2C, and MEF2D, whereas SRF typically remained single-copy (Potthoff and Olson, 2007). Although the MEF2 gene family in animals did not undergo the dramatic expansion seen in land plants, the proliferation of alternative splice forms has greatly increased transcription factor diversity (Theißen *et al*., 1996). According to Ensembl (www.ensembl.org), the four human *MEF2* paralogs collectively generate over 50 transcript isoforms, and extensive alternative splicing has likewise been reported for many other animal *MEF2* genes. In contrast, SRF-type MADS-box genes rarely undergo alternative splicing, as human *SRF*, *Drosophila melanogaster SRF*, *Caenorhabditis elegans SRF*, and yeast *MCM1* and *ARG80* each produce only a single transcript.

Convergently with metazoans, plant MEF2-type genes were recruited into developmental patterning programmes while retaining their ancestral roles in stress responses and reproduction (Fig. 3). After terrestrialization, duplication of the ancestral MEF2-type genes produced the Type I and Type II precursors, enabling their subsequent specialization into diverse gametophytic and sporophytic regulatory networks. This expansion likely facilitated the evolution of more intricate body architectures capable of responding to increasingly variable terrestrial environments. Notably, repeated rounds of duplication and divergence within the Type II lineage may have promoted the shift from a gametophyte-dominant life cycle towards sporophyte dominance in vascular plants, whereas in gametophyte-dominant bryophytes such as mosses and hornworts, Type I genes underwent extensive duplication and diversification. Thus, across both the animal and plant kingdoms, the increase in MEF2-type MADS-box regulatory complexity, through alternative splicing in animals and gene duplication in plants, has been a key driver in the independent evolution of increasingly sophisticated multicellular body plans.

## Conclusion

Among their many regulatory roles, MADS-box transcription factors have key functions in plant reproduction and have been closely linked to the ecological rise and dominance of angiosperms (Kaufmann *et al*., 2005). Understanding how plant MADS-box genes diversified and how their regulatory networks evolved is therefore essential both for explaining the evolutionary success of angiosperms and for guiding agricultural innovation aimed at improving crop productivity.

Flowers and fruits, hallmark innovations of the angiosperm sporophyte, arose through extensive lineage-specific duplication and diversification of Type II (MIKC^C^-type) MADS-box genes. Equally crucial for angiosperm reproduction, M-type genes regulate female gametophyte development and endosperm formation (Masiero *et al*., 2011; Batista *et al*., 2019; Qiu and Köhler, 2022), whereas MIKC-type* genes are required for the development and function of pollen, the male gametophyte (Verelst *et al*., 2007a, b; Adamczyk and Fernandez, 2009; Liu *et al*., 2013). These three MADS-box lineages were already established early in land plant evolution, prior to the divergence of bryophytes and vascular plants. Recent phylogenetic advances place the origin of M-type genes considerably more recent than previously assumed and reveal that they share a closer evolutionary relationship with MIKC*-type genes, together forming the updated Type I clade. Their early function was likely a continuation of the ancestral role of MADS-box TFs in gametophytes, as suggested by expression patterns in charophytic algae, thereby enabling the MIKC^C^-type lineage to undergo repeated rounds of duplication and neofunctionalization in the sporophyte. This partitioning of regulatory functions set the stage for the evolution of increasingly complex sporophytic body plans, promoting the successful terrestrialization of land plants, and ultimately underpinning the remarkable morphological and ecological diversification of angiosperms.
